# Coaching Clinic as a Strategy to Improve Knowledge and Competence of Nurses in Providing Genetic Counseling Interventions among Thalassemia Patients

**DOI:** 10.30476/IJCBNM.2021.92764.1883

**Published:** 2022-01

**Authors:** Henri Setiawan, Suhanda Suhanda, Doni Setiawan

**Affiliations:** 1 Department of Nursing, STIKes Muhammadiyah Ciamis, Ciamis, Indonesia; 2 Department of Medical Laboratory Technology, STIKes Muhammadiyah Ciamis, Ciamis, Indonesia

**DEAR EDITOR**

Knowledge and competence of nurses in providing genetic counseling interventions in Indonesia needs to be improved. ^1^ The reason is, both thalassemia patients and their parents, as the closest caregivers, need comprehensive information about the genetic problems they are experiencing, the impacts that arise, and the therapeutic management that should be done. It is believed that thalassemia patients and caregivers have good and sufficient knowledge, so that they are able to adapt to physical and psychological problems that arise such as worry, anxiety, and depression. ^2^ Unfortunately, the knowledge and competence about genetic counseling intervention is not strong enough for nurses due to several factors such as the lack of integration of genomic nursing in the nursing education curriculum, absence of a genomic nursing collegium or association under the Indonesian National Nurses Association, and most importantly the absence of regulations set forth in the form of standard operating procedures in hospitals and clinics.

One of the efforts that can be make to strengthen the knowledge and competence about genetic counseling is coaching clinic. The learning model with coaching clinic allows the nurses to receive information about basic knowledge of genetic counseling, as well as gain adequate experience from clinical practice. Coaching Clinic can use various learning methods such as Discovery Learning (DL), Small Group Discussion (SGD), Role Play and Simulation (RPS), Collaborative Learning (CL), and Project-Based Learning and Inquiry (PjBL). ^3^

The DL model is used as a strategy to obtain conclusions about the basic concepts of genetic counseling and the role of nurses as counselors through an intuitive process. However, the depth of the intuitive process is limited to the knowledge, literacy, and experience of each nurse. This model needs to be strengthened with the SGD method, so that interactions occur between nurses, making conclusions that are more critical, systematic, and widespread. ^4^ These two methods are appropriate to be used to discuss the pertinent topics such as human genome project, chromosomal aberration, gene mutation on thalassemia, therapy management, and implication of nursing metaparadigm.

The RPS, CL, and PjBL models will be more effectively used to strengthen the experience of nurses in designing a case, overcoming the main problem in the case, preparing a work plan, and providing genetic counseling interventions and evaluation. The scenarios made describe the variety of physical and psychological problems in thalassemia patients and their parents as caregivers who are closest to the patient. Pre-briefing and post-briefing are carried out to ensure nurses understand the context of the problems experienced by thalassemia patients and caregivers. Thus, nurses can develop a detailed and specific genetic counseling intervention plan. ^5^

 The level of knowledge and competence of nurses was measured before and after the Coaching Clinic to capture changes in the outcome target score, using the appropriate instrument. Although it is certain that the favorable score will increase, the success rate of the coaching clinic strategy may not be the same at different times and places. Therefore, outcome measurement is required in this coaching clinic. No matter how strong the level of significance of the influence of the coaching clinic is, the experience of nurses from this coaching clinic has a positive influence on the attitudes and character of nurses to optimize their roles and functions as counselors in providing genetic counseling. ^6^

## ACKNOWLEDGEMENT

We would like to thank the Ministry of Education and Culture of the Republic of Indonesia and the National Research and Innovation Agency.

**Conflict of Interest:** None declared.
